# Editorial: Reproduction in South American Camelids

**DOI:** 10.3389/fvets.2021.722949

**Published:** 2021-08-10

**Authors:** Marcelo Ratto, Marcelo Horacio Miragaya

**Affiliations:** ^1^Instituto de Ciencia Animal, Facultad de Ciencias Veterinarias, Universidad Austral de Chile, Valdivia, Chile; ^2^Facultad de Ciencias Veterinarias, Instituto de Investigación y Tecnología en Reproducción Animal, Universidad de Buenos Aires, Buenos Aires, Argentina

**Keywords:** Llama (Lama glama), alpaca, reproductive biotechnology, theriogenology, andrology

When we proposed this Research Topic to Frontiers in Veterinary Science, we aimed to invite and congregate the best of the ongoing research that expands our current understanding of reproductive biology in South American camelids (SACs). We did not pursue this only as a way to communicate among the scientific community researching in the field but also to share the fascinating aspects of reproduction in these species to a broad audience, including practitioners and colleagues working on education, conservation, and applied reproduction. In this Research Topic, we received contributions that include a mini-review and several original research articles encompassing fundamental aspects of reproductive physiology and applied reproductive biotechnologies.

SACs are species displaying unique reproductive features, many of which still are not understood; examples of these are their induced ovulatory mechanism and laterality of gestation. In this Research Topic, Berland et al. report neuroanatomical aspects of brain GnRH and kisspeptin neurons in female llamas; these neuropeptide-synthesizing cells orchestrate the release of gonadotrophic hormones that control follicle development and ovulation. A second contribution by Gallelli et al. characterize the temporal association between follicular waves and circulating concentrations of estradiol and insulin grow factor-1 in llamas, showing that the rise of both hormones parallels the development of follicular waves. In another study, Bianchi et al. report evidence of dose-dependent estradiol-induced ovulation in llamas, resignifying the potential role of estradiol in the ovulatory mechanism of SACs. In another piece of original research, Norambuena et al. report that alpacas under moderate energy restriction negatively affect body condition score and CL size, but this does not result in significant changes in CL vascularization and progesterone concentrations. Finally, a study by Ratto et al. show that the ovulation laterality and intrauterine embryo location do not induce asymmetrical differences of the mesometrial and endometrial vascularization area of uterine horns during the first 30 days of gestation.

Several pieces of evidence have shown that the beta-nerve growth factor (β-NGF) –which is also implicated in ovulation in SACs– exerts pivotal roles in the reproductive biology of SACs. In this line, Valderrama et al. show that β-NGF enhances the expression of genes involved in angiogenesis and progesterone synthesis as well as progesterone output in preovulatory llama granulosa cells *in vitro*. After conception, more than 90% of the gestations occur in the left uterine horn in SACs; a study conducted by Barraza et al. characterize the bilateral horn gene and immunohistochemical spatial expression of β-NGF and its receptor, TrkA, in the endometrium of non-pregnant and early pregnant alpacas; the gene expression of the angiogenic factor VEGFA and number of vessels was also assessed.

Closing the set of physiological aspects of reproduction in SACs, El Zawam et al. determine serum testosterone concentrations in response to administration of human chorionic gonadotropin (hCG) and its correlation with testicular weight in alpacas of different age.

The lack of understanding of the peculiar reproductive characteristics of SACs has impeded to make substantial improvement of their low reproductive rates. It is essential to improve and develop new strategies that increase reproductive efficiency, and so the development of biotechnologies is necessary to fulfill these objectives. For example, semen cryopreservation has low efficiency, and when artificial insemination is performed with it, poor results are obtained, affecting the advances in genetic progress and selection. Here, we received a set of different studies related to reproductive biotechnologies in female and male SACs. This set opens with a review by Morrell and Abraham about semen collection and handling in SACs. Original research conducted by Zampini, Castro-González et al. report electron microscopy ultrastructural alterations of llama sperm during cooling and freezing, using a conventional camelid semen cryopreservation protocol. Another study by Carretero et al. shows that the air-drying process has a negative effect on llama sperm DNA. Finally, Valdivia et al. report cryopreservation of alpaca testicular tissue and isolated testicular cells.

Several contributions aimed to develop strategies to enhance the performance of cryopreserved semen were received in this Research Topic. In one of these, Sari et al. report that the addition of β-NGF significantly increases the percentage of total motility and vigor of post-refrigerated sperm. Another study by Guillén Palomino et al. evaluate the efficiency of Androcoll-E™ to separate llama sperm from seminal plasma and freezing extender in frozen-thawed semen, demonstrating their separation while preserving the viability, membrane function, and acrosome integrity. In the same area, Bertuzzi et al. compare a non-commercial extender with egg yolk and the commercial extender Andromed® with and without egg yolk for cooling alpaca sperm obtained from diverted deferent ducts. A study by Aisen et al. investigated the effect of whole seminal plasma addition to alpaca spermatozoa after the freezing-thawing-process on dynamic and morphological parameters and an artificial insemination trial. Closing the set of contributions focused on semen cryopreservation, a study conducted by Lutz et al. report a field-efficient technique for cryopreservation of alpaca preimplantation embryos using a modified horse vitrification protocol.

In the subject of female reproductive biotechnology, Zampini, Veiga et al. report the development of a synchronization and superstimulation protocol for embryo donors in llamas showing that a protocol based on GnRHa, PGF2α, and eCG allows a fixed-timed mating without the use of ultrasonography. In another contribution of Zampini, Gallelli et al. using color-Doppler ultrasonography, they show that the uterine blood flow (UBF), but not corpus luteum blood flow (CLBF), may be a useful predictor for early pregnancy diagnosis in llamas 8 days post-mating.

The papers presented in this Research Topic provide new insights into different aspects of reproductive biology of SACs and also demonstrate the efforts of the current research to provide a better understanding of their peculiarities that, in turn, will allow overcoming challenges in applied reproduction in SACs.

We would like to thank all the authors who contributed to this Research Topic and express our gratitude to the editors, reviewers, and staff of Frontiers in Veterinary Science that made possible this Research Topic in Reproduction in South American Camelids. Finally, we also extend our thanks to Frontiers Media for the fee waivers provided which supported some of the contributions presented in this Research Topic.

## Author Contributions

MR and MHM wrote the editorial together. Both authors listed have made a substantial, direct and intellectual contribution to the work, and approved it for publication.

## Conflict of Interest

The authors declare that the research was conducted in the absence of any commercial or financial relationships that could be construed as a potential conflict of interest.

## Publisher's Note

All claims expressed in this article are solely those of the authors and do not necessarily represent those of their affiliated organizations, or those of the publisher, the editors and the reviewers. Any product that may be evaluated in this article, or claim that may be made by its manufacturer, is not guaranteed or endorsed by the publisher.

